# Long-Term Safety of Antifibrotic Drugs in IPF: A Real-World Experience

**DOI:** 10.3390/biomedicines10123229

**Published:** 2022-12-12

**Authors:** Stefano Levra, Giuseppe Guida, Andrea Elio Sprio, Flavio Crosa, Paolo Carlo Ghio, Francesca Bertolini, Vitina Carriero, Carlo Albera, Fabio Luigi Massimo Ricciardolo

**Affiliations:** 1 Department of Clinical and Biological Sciences, University of Turin, 10043 Turin, Italy; 2 Severe Asthma and Rare Lung Disease Unit, San Luigi Gonzaga University Hospital, Orbassano, 10043 Turin, Italy; 3 Department of Medical Sciences, University of Turin, 10124 Turin, Italy; 4 Division of Respiratory Medicine, Cardiovascular and Thoracic Department, AOU Città della Salute e della Scienza di Torino, 10126 Turin, Italy; 5 Institute of Translational Pharmacology, National Research Council (IFT-CNR), Section of Palermo, 90146 Palermo, Italy

**Keywords:** pirfenidone, nintedanib, real-life, idiopathic pulmonary fibrosis

## Abstract

Pirfenidone and nintedanib are the only two drugs approved for the treatment of idiopathic pulmonary fibrosis (IPF). Both proved to be safe and well-tolerated in clinical trials, but real-world data and direct comparisons are scarce. This real-life study explored the safety profile of pirfenidone and nintedanib with a prolonged follow-up. We retrospectively collected clinical status, adverse events (AEs), and treatment changes from IPF patients who had started an antifibrotic treatment at our centre from December 2011 to December 2020, including 192 patients treated with pirfenidone and 89 with nintedanib. The majority of patients in both groups experienced one or more AEs during the follow-up. A higher proportion of AEs in the nintedanib group were effectively treated with behavioural modifications or additional medications compared with the pirfenidone group (52.5% vs. 40.6%, *p* = 0.04). Overall, a difference in the impact of AEs due to nintedanib versus pirfenidone resulted in a lower permanent discontinuation of therapy (8.3% vs. 18.3%, *p* = 0.02), with the latter being associated with a higher risk of drug discontinuation at 48 months after initiation (OR = 2.52, *p* = 0.03). Our study confirms the safety profile of antifibrotic drugs in IPF but highlights that AEs due to nintedanib are usually easier to manage and lead to fewer cases of permanent discontinuation of therapy.

## 1. Introduction

To date, only two drugs have been approved for the treatment of idiopathic pulmonary fibrosis (IPF): pirfenidone and nintedanib [1]. In Italy, pirfenidone has been available since 2011 and nintedanib since 2015. The prescription and reimbursement criteria for both drugs are based on a patient’s age and pulmonary function, specifically forced vital capacity (FVC) and diffusion lung capacity for carbon monoxide (DLco). As in other countries, to be eligible for reimbursement, a patient who starts pirfenidone must be ≤80 years old and have values of FVC percent predicted (FVC%pred) ≥ 50 and DLco percent predicted (DLco%pred) ≥ 35. For nintedanib reimbursement, there are no age limits, but the patient must have values of FVC%pred ≥ 50 and DLco%pred ≥ 30. These criteria are derived from those used in the studies that led to the approval of the two drugs [2,3,4]. The evidence reported in clinical trials highlighted that these two drugs seem to have the same efficacy and safety profile. The two largest trials that have evaluated pirfenidone in IPF patients, CAPACITY and ASCEND, described that the most common adverse events (AEs) that occurred were nausea, dyspepsia, and rash [2,3]. Concerning nintedanib, the INPULSIS 1 and 2 studies observed that gastrointestinal events, particularly diarrhoea, were the most common AEs [4]. Subsequently, similar tolerability profiles were reported in the extension studies, RECAP for pirfenidone and INPULSIS-ON for nintedanib [5,6]. Most studies comparing the safety and tolerability of pirfenidone and nintedanib ended within 2 years after initiation, and long-term data are still uncertain. Moreover, there are no clinical trials in which pirfenidone and nintedanib were directly compared, and real-world data from unselected IPF populations are still scarce. To date, even if presently collected data confirm the tolerability profile of the two antifibrotic drugs, many issues remain unsolved. In fact, drug discontinuation still occurs in a considerable proportion of patients, and first line therapy for the individual patients must be optimised [7].

In this real-life study, we intended to compare in IPF patients the tolerability profile of pirfenidone and nintedanib during an extended follow-up prolonged up to 48 months from the start of the therapy.

## 2. Materials and Methods

### 2.1. Study Design and Participants

**Figure 1:**
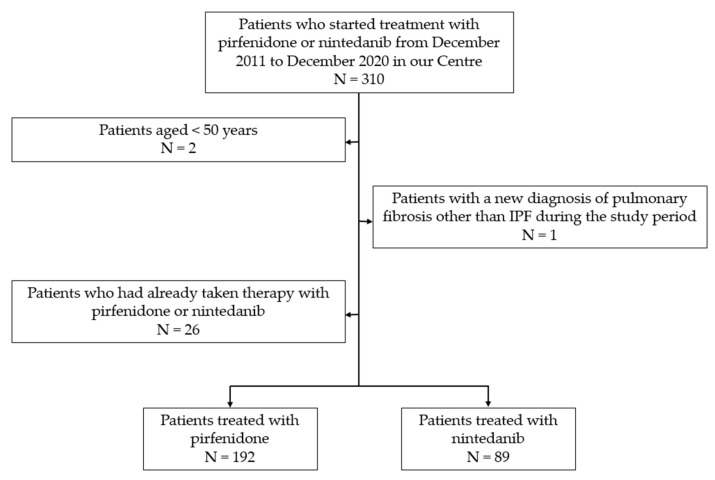
Patient flowchart.

In this observational retrospective single-centre study, 310 outpatients were enrolled. Data were collected from the medical records available at the “Centre of Interstitial and Rare Lung Diseases” of the San Luigi Gonzaga University Hospital (Orbassano, Turin, Italy). The study was conducted according to the principles of the Declaration of Helsinki and approved by the local ethical committee (protocol No. 239/2021). All participants provided written informed consent to participate in this study.Inclusion criteria were an IPF diagnosis confirmed according to the American Thoracic Society (ATS), the European Respiratory Society (ERS), the Japanese Respiratory Society (JRS), and the Latin American Thoracic Association (ALAT) guidelines [8,9] and the initiation of pirfenidone or nintedanib treatment between December 2011 and December 2020. Since both drugs were available, patients decided which drug to take after receiving information on expected efficacy and possible AEs. Patients younger than 50 were excluded from statistical analysis, as well as patients with a new diagnosis of pulmonary fibrosis other than IPF during the study period. Only patients who started the antifibrotic treatment for the first time were included in the study population, while those who had already received pirfenidone or nintedanib were excluded. Of the 310 patients enrolled, 281 were included in the analysis. Of these, 192 were treated with pirfenidone and 89 with nintedanib. The flowchart of patient selection is shown in Figure 1. 

### 2.2. Procedures

Treatment initiation date was defined as the first day when the drug was dispensed by a pharmacy. The date of treatment initiation was considered “time zero” for all longitudinal analyses. Patients on pirfenidone therapy started at a dose of 267 mg three times daily and reached a dose of 801 mg three times daily over three weeks. Patients on nintedanib therapy started with a dose of 100 mg twice daily and switched to a dose of 150 mg twice daily after one month. Each patient was monitored from the beginning of therapy every 3 months until the last available medical record, lung transplant, death, or the 48th month of follow-up, depending on what occurred first. A reassessment of clinical status, AEs of therapy, and liver enzymes was performed at each check with potential therapeutic adjustment. At least twice a year, each patient had pulmonary function tests for FVC and DLco. For this study, we collected data from the annual and semi-annual visits performed between December 2011 and May 2021. The gender–age–physiology (GAP) index was used retrospectively to divide patients into stages of disease severity [10]. 

Additional medications were recommended to all patients to manage gastrointestinal symptoms (i.e., domperidone for nausea and loperamide for diarrhoea). Behavioural modifications were set up to minimise photosensitivity (i.e., avoiding or protection from direct sunlight and to use sunscreen). Permanent or temporary dose reductions and treatment interruptions were allowed to manage persistent adverse events. The reduced dose for nintedanib was 200 mg per day, whereas for pirfenidone, it was equal to the highest tolerated dose below 2403 mg per day. 

### 2.3. Statistical Analysis

Descriptive analyses included means and medians, according to the nature of the variables. As dispersion measurements, the standard deviation (SD) and the interquartile range (IQR) were calculated. Either the unpaired Student’s t-test or Mann–Whitney U-test were used to compare the differences between two groups, while the chi-squared (χ^2^) test was employed to compare frequencies. Results were considered statistically significant for *p* < 0.05. Statistical evaluations were performed with GraphPad Prism 9.0.2 (GraphPad Software, San Diego, CA, USA).

## 3. Results

### 3.1. Baseline Characteristics

The mean observational period was 27 ± 17 and 17 ± 14 months for the pirfenidone and nintedanib groups, respectively. A total of 51 patients completed the follow-up in the pirfenidone group, and 5 in the nintedanib group. The demographic and clinical data of outpatients are reported in Table 1. The pirfenidone-treated group and the nintedanib-treated group were comparable in terms of sex and smoking history. Patients treated with nintedanib were significantly older than those treated with pirfenidone (71.7 vs. 75.1 years, *p* < 0.01). No differences were found in FVC%pred at baseline, but patients treated with nintedanib had significantly lower DLco%pred than those treated with pirfenidone (43.6% vs. 50.6%, *p* < 0.01). Overall, patients initiating nintedanib therapy were significantly more impaired and showed a higher GAP index stage (1.9 vs. 1.6, *p* < 0.01). This led to a greater need for supplemental oxygen use, which was significantly higher in the nintedanib-treated group (15.1% vs. 36% of patients, *p* < 0.01). 

### 3.2. Adverse Events and Therapy Adjustments

Of the 192 patients who started treatment with pirfenidone, 116 (60.4%) had AEs. There were a total of 224 AEs, with an average of 1.93 per patient. Most patients experienced one (n = 49, 42.2%) or two (n = 40, 34.3%) AEs, but 27 experienced three or more (23.2%). The majority of AEs that occurred were gastrointestinal, such as nausea (21.9%), weight loss (12.5%), dyspepsia (11.6%), and loss of appetite (8.9%). Photosensitivity (14.7%) and skin rash (6.7%) were also common. No drug-related fatal events occurred (Table 2). 

Of the 224 AEs due to pirfenidone, 91 (40.6%) were managed with non-pharmacological intervention (e.g., behavioural modifications) or additional medications. The therapy adjustments required for the other AEs (59.4%) are shown in Table 2. AEs that more often resulted in therapy adjustment were nausea, photosensitivity, inappetence, and weight loss. Of these, the most frequent were photosensitivity (5.7% of patients) and nausea (4.7% of patients). Dose reduction was used in 45 patients who experienced an AE (38.8%). Of these, 32 (71.1%) were able to continue antifibrotic therapy. In contrast, temporary cessation was used in 15 patients of those who had an AE (12.9%), and 9 (60%) of them were able to resume therapy with improved tolerance (Table 3). In addition, one patient discontinued therapy because of lack of efficacy during the follow-up period, and three on their own initiative. Active smoking led to a temporary end in three cases, but never to permanent discontinuation. 

In the nintedanib group, 69.6% of patients experienced AEs. The total number of AEs was 120, with a mean of 1.93 per patient. As for pirfenidone, most patients experienced one (n = 29, 46.8%) or two (n = 15, 24.2%) AEs, but 18 experienced three or more (29%). Almost all were gastrointestinal, such as diarrhoea (40.8%), nausea (15.8%), transaminases elevation (14.2%), weight loss (13.3%), dyspepsia (5%), abdominal pain (4.2%), and vomiting (3.3%). No patients had a severe bleeding event or an acute myocardial infarction during the study, and no drug-related fatal events occurred (Table 4).

Behavioural modifications or additional medications were sufficient to manage 63 of the 120 AEs (52.5%). The therapy adjustments required are reported in Table 4. The adverse events that caused therapy adjustment were mainly diarrhoea, liver enzyme abnormalities, nausea, and weight loss. The most frequent of these was diarrhoea (4.5% of patients). No one discontinued therapy due to lack of efficacy. 

Dose reduction was used in a total of 27 patients who experienced an AE (43.5%). Of these, 19 (70.3%) were able to continue antifibrotic therapy. In contrast, the temporary stop was used in 11 patients of those who experienced an AE (17.7%), and 6 (54.5%) of them were able to resume therapy with better tolerance (Table 3). 

**Figure 2:**
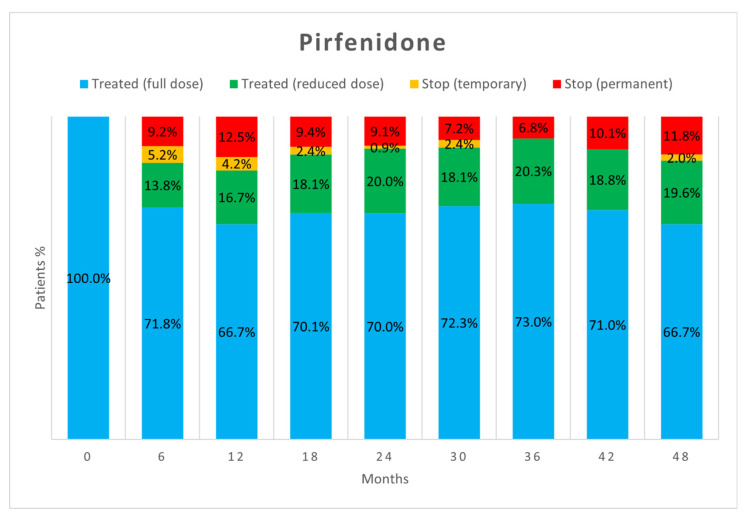
Treatment status of patients in the pirfenidone group at each follow-up visit.

**Figure 3:**
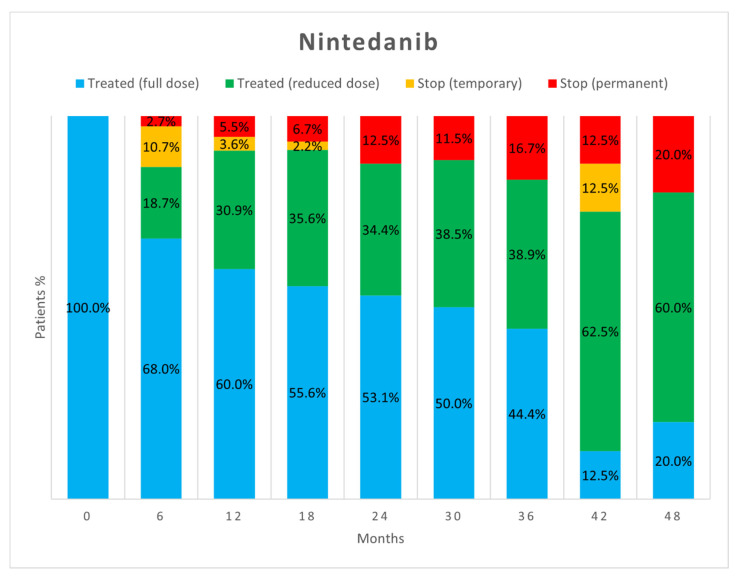
Treatment status of patients in the nintedanib group at each follow-up visit.

Figure 2 and Figure 3 show the percentage of patients who, at each visit, were taking full-dose and reduced-dose therapy as well as the percentage of patients who had temporarily or permanently discontinued therapy. The data only take into account those patients who performed the follow-up visit, while those who missed the visit, were lost at follow-up, or died are not considered.

At the end of the follow-up period, 34 patients (17.7%) had discontinued pirfenidone and 7 (7.8%) had discontinued nintedanib because of AEs. This difference was statistically significant (*p* = 0.03) with an odds ratio of 2.5 (95% CI 1.07–6.22). In both groups, half of the patients discontinued therapy about 6 months after initiation and three-quarters within 1 year. The median time between treatment initiation and permanent discontinuation was 0.52 years for nintedanib (25–75% IQR 0.31–0.97) and 0.53 for pirfenidone (25–75% IQR 0.24–0.90). The percentage of patients who had to discontinue therapy among those who experienced AEs was significantly higher in the pirfenidone group (29.3% vs. 11.3%, *p* < 0.01).

At the start of the treatment, the mean age of patients that discontinued therapy was 74 years in both groups (74.1 for pirfenidone and 74.7 for nintedanib). No significant differences were found in the need for permanent discontinuation (13.5% vs. 6.7%) or dose reduction (27.5% vs. 30.5%) of nintedanib in patients aged 80 years or older (*p* = 0.43 and *p* = 0.99, respectively).

## 4. Discussion

In this retrospective single-centre study, the long-term safety and tolerability of antifibrotic drugs were compared in IPF patients who initiated treatment with pirfenidone (n = 192) or nintedanib (n = 89) in a real-life setting. 

The higher prevalence of men and smokers in our population was in line with expectations [11]. The two groups were comparable in terms of sex, smoking history, and time from diagnosis to treatment initiation. Patients starting nintedanib were older (75.1 vs. 71.7 years, *p* < 0.01) and had a significantly lower DLco% (43.6% vs. 50.6%, *p* < 0.01) than those treated with pirfenidone. This led to a higher average GAP index stage (1.9 vs. 1.6, *p* < 0.01) and a more frequent need for supplemental oxygen (36% vs. 15.1% of patients, *p* < 0.01). These differences can be explained by diversity in the prescription and reimbursement modalities for the two drugs in Italy, which leads to the prescription of nintedanib in patients who are not candidates for pirfenidone because of age or impaired DLco%. This is not the first Italian study reporting a higher mean age and a lower DLco% in nintedanib patients at the time of prescription of therapy [12,13].

Our study provides further evidence that both antifibrotic drugs are safe, although most patients develop AEs. The percentage of patients who developed AEs appeared higher in the nintedanib group (69.7% vs. 60.4%), but this difference did not reach statistical significance (*p* = 0.15). As reported before [14], patients who manifested AEs often simultaneously developed multiple AEs, with a mean of 1.93 per patient for both drugs. 

The AE profile was in line with that of previous studies [6,15,16,17,18]. Most AEs were gastrointestinal for both drugs, with a clear prevalence of nausea for pirfenidone and diarrhoea for nintedanib. Pirfenidone often resulted in cutaneous AEs. With the exception of weight loss, all other AEs were less frequent in our study than in previous clinical trials with pirfenidone and nintedanib [2,3,4]. This could be due to the real-world nature of our study, the peculiar demographic and geographic characteristics of the patients, and the propensity of managing mild AEs by general practitioner intervention. 

As already reported in the literature [17], about half of AEs did not require adjustments to antifibrotic therapy as they were effectively treated with behavioural modifications or additional medications. However, in our study, a difference emerged between the two drugs as a significantly higher proportion of AEs due to nintedanib resulted in no change in antifibrotic therapy (52.5% vs. 40.6%, *p* = 0.04). When adjustment of therapy was necessary, the most commonly used strategy was dose reduction followed by temporary discontinuation. Both strategies proved to be useful in allowing therapies to continue. Dose reduction allowed continuation of antifibrotic therapy in more patients than temporary suspension, but no significant difference appeared between the two strategies (*p* = 0.23). Dose reduction seems to be important especially for nintedanib, as the percentage of patients on reduced-dose therapy increased over time. 

A significantly lower rate of AEs due to nintedanib led to permanent discontinuation of therapy (8.3% vs. 18.3%, *p* = 0.02). This may be due to the essentially gastrointestinal nature of nintedanib AEs and the availability of effective therapies that can be added. As reported previously [6,14], although diarrhoea and nausea are very common with nintedanib, they only rarely lead to discontinuation of therapy. The AEs of pirfenidone, on the other hand, are sometimes more difficult to treat [18]. This is especially true for the cutaneous ones, which may be difficult to manage despite well-established recommendations and behavioural changes [19]. Indeed, this is not the first study in which cutaneous AEs were the most commonly linked to pirfenidone discontinuation, although they are less frequent than nausea [5,15]. 

Comprehensively, pirfenidone was associated with a higher risk of drug discontinuation than nintedanib at 48 months from the start (OR = 2.52; 95% CI 1.07–6.22). Compared with clinical trials [2,3,4], the discontinuation rate was slightly higher for pirfenidone and significantly lower for nintedanib. Real-life studies directly comparing discontinuation of therapy between the two drugs are scarce, and most ended within 2 years after initiation. However, the discontinuation rate in our study was lower for both pirfenidone and nintedanib in comparison with the literature [14,17,18,20,21]. This may be, at least in part, because we had only one patient drop out for lack of efficacy, and we excluded from the count those patients who had died on therapy. The results of real-life studies are often conflicting: some are in favour of pirfenidone, and others in favour of nintedanib [21,22,23]. Variables that have been associated with an increased risk of treatment discontinuation are older age [17,18,23], female sex [23], FVC%pred < 60% [24], and use of supplemental oxygen [23]. In our study, the risk of drug discontinuation was lower in the nintedanib-treated group, although patients had a higher mean age and a greater need for supplemental oxygen. This supports the better tolerability of nintedanib in terms of requirement of treatment discontinuation, provided that all possible strategies are adopted to control AEs. In contrast to the study of Harari et al. [25], no significant differences emerged in the need for permanent discontinuation or dose reduction of nintedanib in patients aged 80 years or older, although a similar trend may be seen regarding temporary discontinuation.

Our study confirms that most of the AEs leading to treatment discontinuation usually occur in the first 6 months of therapy for both drugs [5,6,26,27]. Patients who tolerate full-dose pirfenidone therapy after this time will likely tolerate it thereafter. In contrast, tolerance to nintedanib seems to vary even many months after initiation, and dose reduction may be necessary at any time. This strategy usually allows the drug to be tolerated and treatment to continue, resulting in a lower percentage of patients having to discontinue therapy. Importantly, the dose reduction made to manage AEs seems to not reduce the benefits of treatment in decreasing lung function decline [4,6,27]. 

A clear limitation of this study is its retrospective and monocentric nature. Another limitation is the selection bias due to the reimbursement modalities in Italy, which often leads to the prescription of nintedanib in patients who are not candidates for pirfenidone because of age or impaired DLco%. Another major limitation is the decreasing patient numbers over time, particularly in the nintedanib group. The main reasons for the limited follow-up were death and the patient’s spontaneous decision to interrupt it, plus, of course, discontinuation due to AEs. In some cases, the short follow-up was determined by lung transplantation. Nintedanib has been available since 2015, so the coronavirus-19 (COVID-19) pandemic strongly influenced the last part of the follow-up in this group. Many patients postponed or cancelled appointments due to the pandemic, but none stopped treatment. Such a result was achieved through close cooperation with pharmacies and regular communication with patients by telephone or email. An approach of this type, which has also been adopted in other centres [28], has been shown to be useful in the follow-up and management of patients with interstitial lung disease during the pandemic and may also be useful after it ends. 

## 5. Conclusions

In conclusion, our study confirms that pirfenidone and nintedanib are generally well-tolerated, although AEs are common with both. AEs caused by nintedanib might be more frequent, but they are more easily manageable with behavioural modification or additional medications and less often lead to definitive discontinuation of therapy. Thus, the risk of treatment discontinuation appeared higher with pirfenidone, although patients on nintedanib were significantly older and had a greater need for supplemental oxygen. When necessary, definitive discontinuation of therapy occurs in most cases within the first 6 to 12 months from the start. Both dose reduction and temporary discontinuation are effective strategies in allowing therapy to continue, but prevention and treatment of AEs, especially diarrhoea, seem to be crucial. Further real-world studies are needed to confirm our results in a real-world setting. A greater knowledge of predictive factors of response and tolerance to therapies will certainly lead to a choice more tailored to individual patient characteristics, but in the meantime, the initial drug choice should be primarily based on comorbidities, concomitant medications, possible AEs, and patient preferences. The standardisation of prescription and reimbursement modalities of the two drugs seems urgent and necessary to guarantee the same therapeutic possibilities to all patients.

## Figures and Tables

**Table 1:** Baseline characteristics of the patients treated with pirfenidone and nintedanib.

Characteristics	Patients Treated with Pirfenidone (n = 192)	Patients treated with Nintedanib (n = 89)	*p*-Value
Age at treatment initiation, years Mean (SD) Median (IQR) Minimum–maximum	71.7 (5.9) 73 (68–76) 53–80	75.1 (7.1) 76 (70–81) 57–87	<0.01
Sex, n (%) Male Female	157 (81.8%) 35 (18.2%)	72 (80.9%) 17 (19.1%)	0.87
Smoking habit, n (%) Smoker/ex-smoker Non-smoker	137 (71.3%) 55 (28.7%)	65 (73%) 24 (27%)	0.99
Time from diagnosis to treatment initiation, months Mean (SD)	5.9 (17.2)	4.9 (15.9)	0.64
FVC at baseline, % of predicted value Mean (SD)	82.4 (18.5)	80.5 (18.7)	0.99
DLco at baseline, % of predicted value Mean (SD)	50.6 (13)	43.6 (15.4)	<0.01
GAP index, stage Mean (SD)	1.6 (0.5)	1.9 (0.6)	<0.01
Use of supplemental oxygen at baseline, n (%) No Yes	163 (84.9%) 29 (15.1%)	57 (64%) 32 (36%)	<0.01

SD, standard deviation; IQR, and the interquartile range; FVC, forced vital capacity; DLco, diffusion lung capacity for carbon monoxide; GAP, gender–age–physiology.

**Table 2:** Frequency of AEs with pirfenidone and related therapy adjustment. Each AE may have undergone multiple therapy adjustments, and each therapy adjustment may have been due to multiple AEs.

Adverse Event	Adverse Events, n (%)	Patients Experiencing AEs (%)	Required Therapy Adjustments, n (%)		
			Dose Reduction	Temporary Discontinuation	Permanent Discontinuation
Appetite loss	20 (8.9%)	10.4%	14 (70%)	5 (25%)	3 (15%)
Diarrhoea	5 (2.2%)	2.6%	-	-	-
Dizziness	9 (4%)	4.6%	2 (22.2%)	1 (11.1%)	2 (22.2%)
Dyspepsia	26 (11.6%)	13.5%	6 (23.1%)	1 (3.8%)	2 (7.7%)
Fatigue	6 (2.7%)	3.1%	2 (33.3%)	1 (16.7%)	2 (33.3%)
Lethargy	1 (0.4%)	0.5%	-	-	-
Nausea	49 (21.9%)	25.5%	18 (36.7%)	3 (6.1%)	8 (16.3%)
Photosensitivity	33 (14.7%)	17.1%	11 (33.3%)	5 (15.2%)	11 (33.3%)
Skin rash	15 (6.7%)	7.8%	3 (20%)	2 (13.3%)	4 (26.7%)
Taste change	5 (2.2%)	2.6%	-	-	-
Liver toxicity	11 (4.9%)	5.7%	4 (36.4%)	-	2 (18.2%)
Vomiting	5 (2.2%)	2.6%	3 (60%)	-	1 (20%)
Weight loss	28 (12.5%)	14.5%	8 (28.6%)	3 (10.7%)	5 (17.9%)
Others	11 (4.9%)	5.6%	-	-	1 (9.1%)
Total	224 (100%)	-	71 (31.7%)	21 (9.4%)	41 (18.3%)

AE, adverse event.

**Table 3:** Therapy adjustments in patients with AEs. Each patient may have undergone multiple therapy adjustments.

Therapy Adjustments Due to AEs	Patients with AEs in the Pirfenidone Group (n = 116)	Patients with AEs in the Nintedanib Group (n = 62)	*p*-Value
No required, n (%)	62 (53.4)	29 (46.8)	0.43
Dose reduction, n (%) Used Effective for treatment continuation	45 (38.8) 32 (71.1)	27 (43.5) 19 (70.4)	0.63 0.99
Temporary discontinuation, n (%) Used Effective for treatment continuation	15 (12.9) 9 (60.0)	11 (17.7) 6 (54.5)	0.38 0.99
Permanent discontinuation, n (%)	34 (29.3)	7 (11.3)	0.01

AE, adverse event.

**Table 4:** Frequency of AEs with nintedanib and related therapy adjustment. Each AE may have undergone multiple therapy adjustments, and each therapy adjustment may have been due to multiple AEs.

Adverse Events	Adverse Events, n (%)	Patients Experiencing AEs (%)	Required Therapy Adjustments, n (%)		
			Dose Reduction	Temporary Discontinuation	Permanent Discontinuation
Abdominal pain	5 (4.1%)	5.6%	-	-	-
Appetite loss	2 (1.7%)	2.2%	-	1 (50%)	1 (50%)
Diarrhoea	49 (40.8%)	55.1%	15 (30.6%)	5 (10.2%)	4 (8.2%)
Dyspepsia	6 (5%)	6.7%	3 (50%)	-	-
Nausea	19 (15.8%)	21.3%	5 (26.3%)	1 (5.3%)	2 (10.5%)
Transaminases elevation	17 (14.2%)	19.1%	8 (47.1%)	3 (17.6%)	1 (5.9%)
Vomiting	4 (3.3%)	4.5%	-	-	-
Weight loss	16 (13.3%)	18%	4 (25%)	2 (12.5%)	2 (12.5%)
Others	2 (1.7%)	2.2%	-	-	-
Total	120 (100%)	-	35 (29.2%)	12 (10%)	10 (8.3%)

AE, adverse event.

## Data Availability

Data available on request from the authors.
